# Analysing the mutational status of *adenomatous polyposis coli* (*APC*) gene in breast cancer

**DOI:** 10.1186/s12935-016-0297-2

**Published:** 2016-03-28

**Authors:** Ya-Sian Chang, Chien-Yu Lin, Shu-Fen Yang, Cheng-Mao Ho, Jan-Gowth Chang

**Affiliations:** Epigenome Research Center, China Medical University Hospital, 2 Yuh-Der Road, Taichung, 404 Taiwan; Department of Laboratory Medicine, China Medical University Hospital, Taichung, Taiwan; School of Medicine, China Medical University, Taichung, Taiwan

**Keywords:** HRM, *APC*, Breast cancer, Direct DNA sequencing

## Abstract

**Background:**

Breast cancer is a heterogeneous disorder for which the underlying genetic basis remains unclear. We developed a method for identifying *adenomatous polyposis coli* (*APC*) mutations and we evaluated the possible association between *APC* genetic variants and breast cancer susceptibility.

**Methods:**

Genomic DNA was extracted from tumor and matched peripheral blood samples collected from 89 breast cancer patients and from peripheral blood samples collected from 50 controls. All samples were tested for mutations in exons 1–14 and the mutation cluster region of exon 15 by HRM analysis. All mutations were confirmed by direct DNA sequencing.

**Results:**

We identified a new single nucleotide polymorphism (SNP), c.465A>G (K155K), in exon 4 and seven known SNPs: c.573T>C (Y191Y) in exon 5, c.1005A>G (L335L) in exon 9, c.1458T>C (Y486Y) and c.1488A>T (T496T) in exon 11, c.1635G>A (A545A) in exon 13, and c.4479G>A (T1493T) and c.5465T>A (V1822D) in exon 15. The following alterations were found in 2, 1, 2, and 1 patients, respectively: c.465A>G, c.573T>C, c.1005A>G, and c.1488A>T. There was no observed association between breast cancer risk and any of these *APC* SNPs.

**Conclusions:**

*APC* mutations occur at a low frequency in Taiwanese breast cancer cases. HRM analysis is a powerful method for the detection of *APC* mutations in breast.

**Electronic supplementary material:**

The online version of this article (doi:10.1186/s12935-016-0297-2) contains supplementary material, which is available to authorized users.

## Background

Breast cancer is a common malignancy and the leading cause of death from cancer among females in economically developing countries, accounting for 23 % (1.38 million) of all new cancer cases and 14 % (458,400) of all cancer deaths in 2008 [1]. The incidence of breast cancer has been increasing in Asia [2, 3]; moreover, breast cancer in Asians occurs at a younger age than in Caucasians. To date, treatment planning for breast cancer patients has been based on a histologic analysis of the primary tumor and the expression of molecular markers such as estrogen receptor (ER), progesterone receptor (PR), and human epidermal growth factor receptor 2 (HER2). Combinations of these markers can be used to further subtype the tumor and guide treatment planning, including luminal A (ER+ and/or PR+; HER2−), luminal B (ER+ and/or PR+; HER2+), basal-like (ER−, PR−, and HER2−), and HER2-enriched (ER−, PR−, and HER2+) markers [4]. Although breast cancer survival has improved significantly within the last few decades, the need for new therapeutic strategies aimed at specific tumorigenic cells and their key oncogenic pathways has become obvious.

Like most cancers, the development of breast cancer is the result of accumulated mutations in oncogenes and tumor suppressor genes. These genes are involved in cell proliferation, growth, differentiation, survival, apoptosis, and DNA repair [5, 6]. A large number of well-established cancer genes have been implicated in breast cancer development, including *BRCAl*, *RB1*, *TP53*, *PTEN*, *AKT1*, *CDH1*, *GATA3*, and *PIK3CA*. Other groups of genes responsible for signal transduction including *APC*, *ARID1A*, *ARID2*, *ASXL1*, *BAP1*, *KRAS*, *MAP2K4*, *MLL2*, *MLL3*, *NF1*, *SETD2*, *SF3B1*, *SMAD4*, and *STK11*, have also been found to contain somatic mutations in tumors. In addition, new cancer genes that had not previously been implicated in the development of breast cancer have been found to be mutated, including *ARID1B*, *CASP8*, *MAP3K1*, *MAP3K13*, *NCOR1*, *SMARCD1*, and *CDKN1B* [7, 8].

*APC*, which was cloned in 1991, is located at chromosome 5q21-22 and contains 15 exons. Its tumor-suppressing activity is thought to be based on regulation of the intracellular level of beta-catenin within the Wingless/Wnt signal transduction pathway [9]. In the absence of Wnts, casein kinase 1 and glycogen synthase kinase-3-bata phosphorylate beta-catenin, causing the ubiquitination and subsequent degradation of beta-catenin by the 26S proteasome. Conversely, when Wnt proteins are secreted from cells, the phosphorylation and degradation of beta-catenin is blocked, leading to its accumulation. The stabilized beta-catenin then translocates to the nucleus, where it binds with T-cell factor/lymphoid enhancer-binding factor-1 to induce the expression of downstream target genes. The mutation of *APC* may cause constitutive stimulation of the Wingless/Wnt signal transduction pathway, promoting the accumulation of beta-catenin in the cytoplasm and leading to aberrant cellular proliferation. Therefore, APC is a negative regulator of the Wingless/Wnt signal transduction pathway. The Wingless/Wnt signal transduction pathway may represent a novel therapeutic target for the treatment of triple-negative breast cancer (ER, PR, and HER 2-negative breast cancer) [10]. In this regard, mutations in *APC* in patients with breast cancer are of particular interest.

*APC* mutations are a major contributing factor to colorectal cancer. In breast cancer patients, the overall *APC* mutational rate ranges from 0.4 to 18 % [11, 12]. We used high-resolution melting (HRM) analysis and direct sequencing to screen exons 1–14 and part of exon 15, including the mutation cluster region (MCR) (codons 1286–1513), of *APC* in breast cancer tissues. The aim of this study was to understand the *APC* mutation status in a Taiwanese cohort of breast cancer patients.

## Results

### HRM analysis of *APC*

The clinical characteristics of the breast cancer patients enrolled in this study, including mean age, age range, and receptor status, are presented in Table 1.

**Table 1:** Clinical characteristics of the breast cancer patients

Characteristics	No	Percentage
Total patients	89	
Age		
mean	53	
range	33–85	
TNM stage^a^		
0	0	0
I	8	11
IIa/IIb	32	44
III/IV	32	44
ER-positive	51	58
PR-positive	71	81
HER2-positive	29	33

^a^TNM stage based on the sixth edition of the American Joint Committee on Cancer (AJCC) Cancer Staging Manual (2002)

**Fig. 1:**
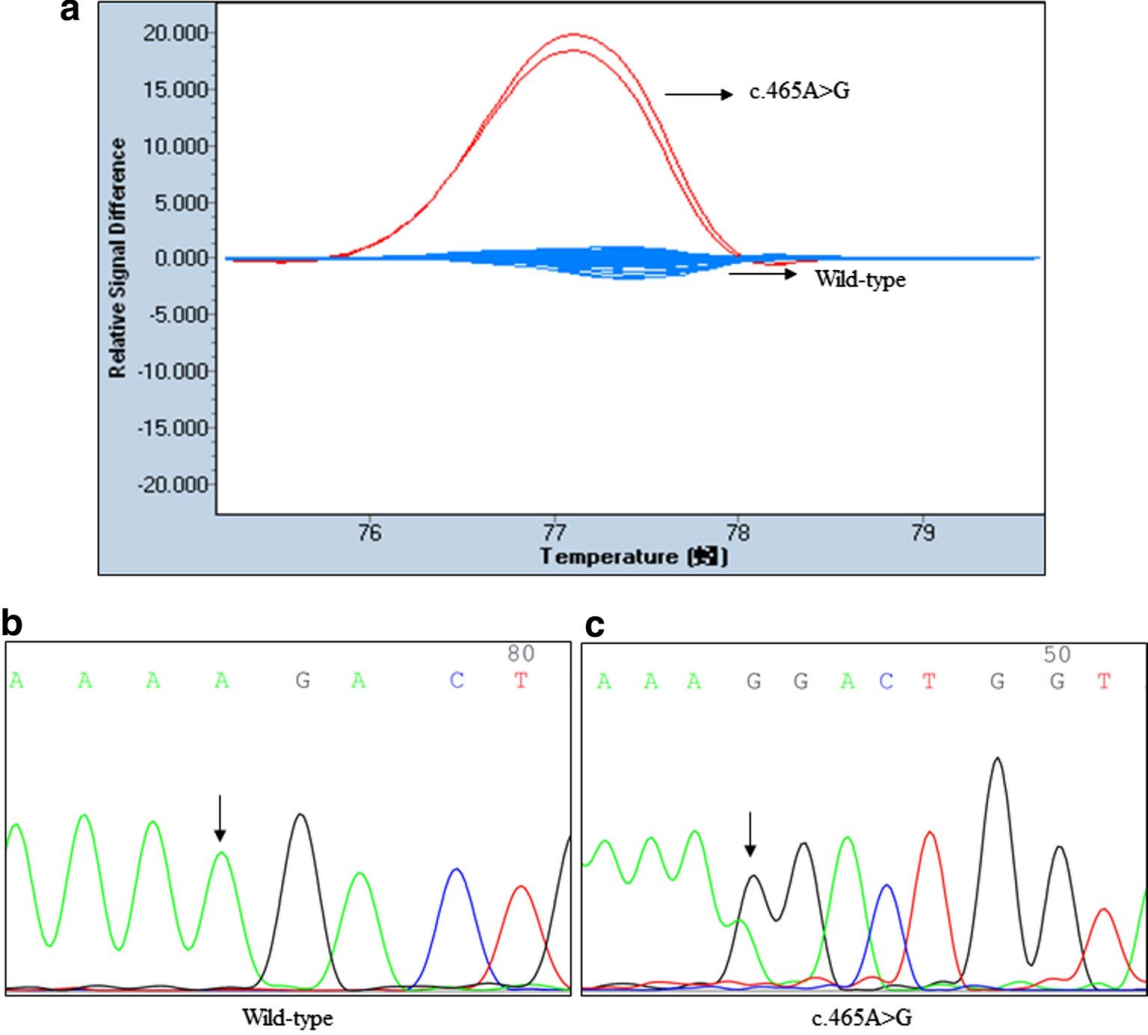
HRM assays and sequence traces for *APC* exon 4. **a** Difference plot showed two different melting profiles, wild-type (WT) samples was *blue*, mutation in other *colors*. Direct DNA sequencing confirmed the **b** WT and the presence of the *APC* exon 4 mutation: **c** c.465A>G

The *APC* gene mutations identified in the 89 patients and 50 controls by HRM analysis are summarized in Table 2. Eight single-nucleotide alterations were detected in the cancerous tissues of the patients, including c.465A>G in exon 4 (Fig. 1), c.573T>C in exon 5, c.1005A>G in exon 9, c.1458T>C and c.1488A>T in exon 11, c.1635G>A in exon 13, and c.4479G>A and c.5465T>A in exon 15 (Additional file 1: Figure S4, S8, S10, S12 and S14). HRM analysis of exons 1–3, 6–8, 10, 12, and 14 failed to identify mutations or polymorphisms in these regions (Additional file 1: Figure S1-S3, S5-S7, S9, S11 and S13). The frequencies of the SNPs in the controls were 0 % for c.465A>G, c.573T>C, and c.1488A>T. The frequencies for c.1005A>G, c.1458C, c.1635A, c.4479A, and c.5465A were 1, 67, 85, 85, and 94 %, respectively, and therefore similar to those in the malignant tissues.

**Table 2:** Single nucleotide alterations in *APC* identified in breast cancers (n = 89) and controls (n = 50) using high resolution melting analysis

Position in gene	Nucleotide location, cDNA (from start codon)	Amino acid location	dbSNP identifier	Han Chinese in Bejing, China (1000 genomes project data) (%)	Cases n = 178 alleles (%)	Controls n = 100 alleles (%)	P value
Exon 4	c.465A>G	K155K					
	Allele A				176 (98.88)	100 (100)	0.5377^*^
	Allele G				2 (1.12)	0 (0)	
Exon 5	c.573T>C	Y191Y	rs185154886				
	Allele T			100	177 (99.44)	100 (100)	1.000^*^
	Allele C			0	1 (0.56)	0 (0)	
Exon 9	c.1005A>G	L335L	rs3797704				
	Allele A			100	176 (98.88)	99 (99)	1.000^*^
	Allele G			0	2 (1.12)	1 (1)	
Exon 11	c.1458T>C	Y486Y	rs2229992				
	Allele T			32.52	52 (29.2)	33 (33)	0.511
	Allele C			67.48	126 (70.8)	67 (67)	
Exon 11	c.1488A>T	T496T	rs9282599				
	Allele A			99.51	177 (99.44)	100 (100)	1.000^*^
	Allele T			0.49	1 (0.56)	0 (0)	
Exon 13	c.1635G>A	A545A	rs351771				
	Allele G			18.93	29 (16.3)	15 (15)	0.777
	Allele A			81.07	149 (83.7)	85 (85)	
Exon 15	c.4479G>A	T1493T	rs41115				
	Allele G			18.93	24 (13.5)	15 (15)	0.727
	Allele A			81.07	154 (86.5)	85 (85)	
Exon 15	c.5465T>A	V1822D	rs459552				
	Allele T			9.22	14 (7.9)	6 (6.0)	0.564
	Allele A			90.78	164 (92.1)	94 (94.0)	

^*^ P value by Fisher’s exact test when the cell expectation was less than five

An evaluation of the relationship between each *APC* SNP and breast cancer risk (Table 2) did not show an association with any of the SNPs at any of the loci.

### Concordance of SNPs in paired tumor and blood samples

To determine whether c.465A>G, c.573T>C, c.1005A>G, and c.1488A>T were somatic or germline abnormalities, we investigated the concordance between genotypes from all 89 paired tumor and blood samples. The data indicated a 97.75 % (87/89) concordance between tumor and blood samples, except for a discrepancy in two samples for c.573T>C and c.1488A>T, in which the mutations were somatic and were detected in the tumor tissues but not in the blood sample.

## Discussion

Mutations in *APC* have been found in several cancers, including colorectal [13], pancreatic [14], and hepatocellular [15], but they are reported to be much rarer in breast cancer. The first report of *APC* mutations in breast cancer was published by Kashiwaba et al. [16], who detected a mutation rate of 6 % (2 of 31) in primary breast carcinoma samples using PCR followed by restriction fragment length polymorphism (RFLP) and single-strand conformation polymorphism (SSCP) analyses. Sorlie et al. [11] studied mutations in exon 15 using a protein truncation test (PTT). They reported a frameshift mutation at nucleotide 4678 in one patient (1 of 227). Furuuchi et al. [12] screened almost the entire coding region using a yeast-based assay. They reported a mutation rate of 18 % (13 of 70) in primary breast cancers; the mutations included five nonsense mutations, five single base pair deletions, and five single base pair insertions. Until now, their study had the highest reported mutation rate in breast cancer. Hayes et al. [17] analyzed exon 15 in 27 metaplastic carcinomas by direct DNA sequencing and found one mutation in two samples (7.4 %). Kan et al. [18] used mismatch repair detection (MRD) technology to identify somatic mutations in 441 tumors, including 183 breast cancers. They found three mutations in three breast tissue samples (1.6 %). Stephens et al. [8] used whole exome sequencing to detect somatic alterations in 100 patients. They found two mutations in two breast samples (2 %). The above-mentioned mutations were unique. In the present study, among the eight *APC* SNPs detected, seven were silent in the 89 breast cancer samples. Of those, five were also found in the control population, one was present in both blood and cancerous tissue, and two (c.573T>C and c.1488A>T) were somatic mutations detected in cancerous tissues only and not in patients’ blood cells. These results suggest that *APC* does not play an important role in the development of breast cancer in the Taiwanese population, although the *APC* mutation rate was higher in breast cancer patients than in controls. However, a limitation of our study was that we did not comprehensively evaluate all of the exons in the control samples.

The methods typically used to detect *APC* mutations have some weakness. PCR-RFLP and PCR-SSCP are simple to perform but require gel electrophoresis to separate the PCR amplicons; they are therefore not suitable for investigating large genes with many exons, such as *APC*, or a large number of samples. In addition, PCR-RFLP can only detect known mutations. PTT requires the use of radioactive labeling for protein detection, after which the DNA from samples with positive PTT results is sequenced. The yeast colorimetric assay consists of four steps: PCR, homologous recombination, fusion protein expression, and a colorimetric assay. The MRD method is a robust and cost-effective, but it is not efficient in detecting insertions/deletions. Exome- or *APC*-specific target sequencing using next-generation sequencing requires expensive equipment and bioinformatic analysis; its clinical application is therefore limited.

Compared with the above-mentioned molecular diagnostic methods, HRM analysis is relatively cost-effective and technically feasible. Although HRM analysis represents the next generation of mutation scanning technology, it also has several limitations [19, 20]. For example, many homozygous variants and small insertions/deletions are more difficult to detect by HRM analysis and may produce only small differences in melting temperature (TM). This will affect the accuracy of genotyping which depends on the temperature resolution capability of the instruction. Instrument resolution is of particular concern in the case of thermal block instruments with 96 or 384 wells. In this study, we failed to identify the substitution of a single nucleotide in exons 11 (c.1458T>C), 13 (1635G>A), and 15 (c.4479G>A and c.5465T>A) by HRM analysis because homozygotes could not be distinguished. To solve this problem, homozygous samples were mixed with a known genotype before HRM analysis. When an equal amount of wild-type DNA was added, the wild-type samples remained the same in the normalized and temperature-shifted difference plots, while the homozygous mutant samples exhibited heteroduplex formation; however, this approach doubled the amount of work.

Although HRM analysis could be used to identify heterozygotes, different heterozygotes may produce similar melting curves to each other. In this study, the exon 11 amplicon was found to contain one SNP, c.1458T>C (p.Y486Y; rs2229992) and one mutation, c.1488A>T (p.T496T; rs9282599). Our HRM analysis was successful in distinguishing variant-specific heterozygosity. Tindall et al. [21] also demonstrated that different heterozygotes were distinguishable by HRM analysis. Unfortunately, HRM analysis is unable to identify which codon is mutated; therefore, direct DNA sequencing should be performed to confirm the position of the mutation when different heterozygotes produce highly similar melting curves. In addition, HRM analysis cannot detect mutations surrounding an entire exon if the exon is too large or if entire exons and genes have been deleted. This would require the design of primer pairs that surround the entire exon. Amplicon length influences the sensitivity of HRM analysis, with amplicons of 100–300 base pairs considered to be optimal. The shorter the amplicon, the greater the Tm differences among genotypes and therefore the better the differentiation between mutant and non-mutant samples. Moreover, long amplicons may contain more melting domains, leading to rather complex melting profiles. Factors that affect the melting behavior of double-stranded (ds) DNA include the MgCl_2_ concentration and the quality and quantity of the DNA. The amounts of DNA in the samples should not differ significantly, in order to achieve similar threshold cycles.

The four *APC* SNPs (c.1458T>C, c.1635G>A, c.4479G>A, and c.5465T>A) had a minor allele frequency of 29.21, 16.29, 13.48, and 7.87 %, respectively. The allele frequencies were similar to those seen in the phase 3 data of the 1000 Genomes Project (Table 2). This is the first investigation of the association between SNPs in *APC* and breast cancer. Previous studies have addressed the association between SNPs in *APC* and colorectal cancer risk [22, 23]. c.3920T>A is the most common *APC* variant described in breast cancer. Previous studies investigated the association between this variant and susceptibility to breast cancer among Ashkenazi Jews [24, 25]; however, no significant contribution to breast cancer susceptibility was found for the four SNPs. However, large-scale studies are needed to validate these findings.

## Conclusions

Our results indicate that the prevalence of *APC* mutations in Taiwanese breast cancer patients is rare, similar to other populations. It can thus be concluded that, at least in these patients, the *APC* gene does not play an important role in the oncogenesis of breast cancer. We also identified two somatic *APC* mutations. HRM analysis is a rapid and cost-effective method for the mutational analysis of *APC*.

## Methods

### Sample preparation and DNA isolation

Breast tissue samples and matched peripheral blood samples from 89 breast cancer patients who had undergone surgery at China Medical University Hospital (Taichung City, Taiwan) from 2003 to 2009 were included in this study. The tissues were frozen immediately after surgical resection and stored in liquid nitrogen until DNA extraction. As controls, 50 healthy individuals were also recruited. Genomic DNA was extracted using a commercially available kit (GE Healthcare, Little Chalfont, UK). The quality of the isolated genomic DNA was assessed using agarose gel electrophoresis, and the concentration was determined using a Nano-Drop-1000 spectrophotometer (Nano-Drop Technologies Inc., Wilmington, DE, USA). This study was approved by the Institutional Review Board of the China Medical University.

### HRM analysis

PCR and a melting analysis of *APC* mutations were performed using the LightCycler^®^ 480, a real-time PCR machine with HRM and 96/384 well capacity. The primers used in this study were previously described [26] and were of standard molecular biology quality (Protech Technology Enterprise Co., Ltd., Taipen City, Taiwan). Controls samples were tested for mutations in exons 4, 5, 9, 11, and 13, and the MCR of exon 15 using HRM analysis. PCR was performed with ~10 ng of DNA and 0.25 μM each of the relevant forward and reverse primers in a total reaction volume of 10 μl. Each reaction consisted of 5 μl of LightCycler^®^ 480 high-resolution melting master reagent (Reference 04909631001; Roche Diagnostics, Basel, Switzerland) and 2.5 mM MgCl_2._ PCR was performed under the following conditions: 95 °C for 10 min, followed by 45 cycles of 95 °C for 15 s, 60 °C for 15 s, and 72 °C for 15 s, with a melting program at 95 °C for 1 min, 40 °C for 1 min, and 60–90 °C (25 acquisitions/°C).

Discrimination of two homozygotes was achieved by generating artificial heterozygotes. Before the HRM analysis, the two homozygous genotypes (1458T vs. 1458C, 1635G vs. 1635A, 4479G vs. 4479A and 5465T vs. 5465A) were mixed with a known genotype (homozygous CC DNA, homozygous AA DNA, homozygous AA DNA and homozygous AA DNA, respectively).

Upon completion of the run, the samples were analyzed using the gene scanning software (version 1.5, Roche Diagnostics), supplied with the LightCycler^®^ 480. The melting curve analysis included: the normalization of melting curves equal to 100 % of the initial fluorescence and to 0 % of the remaining fluorescence. The temperature axis of the normalized melting curves was shifted such that the entire dsDNA was completely denatured. Difference plots were obtained by subtracting the curves of the wild-type and mutant DNAs and were used to cluster the samples into groups.

### Direct DNA sequencing

To confirm the results of the HRM analysis, the same amplicons were sequenced. The PCR products were cleaned with a PCR-M™ clean-up system (Viogen, Sunnyvale, CA, USA). Sequencing reactions were set up using a BigDye Terminator, version 3.1, cycle sequencing kit (Applied Biosystems, Foster City, CA, USA). BigDye^®^ XTerminator™ (Applied Biosystems) was used to purify the reaction products. Sequencing analyses were carried out on an ABI Prism 3130 genetic analyzer (Applied Biosystems).

### Statistical analysis

All statistical analyses were carried out using the statistical software program SPSS 17.0 for Windows (SPSS, Inc., Chicago, IL, USA). Differences in the genotypes of breast cancer patients vs. controls were evaluated using a χ^2^ test. Fisher’s exact test was used when the expected number in any cell was <5. A P value of <0.05 was considered to indicate statistical significance.

## Additional file

10.1186/s12935-016-0297-2 HRM assays for *APC* exons 1, 2, 3 and 5-15.
