# Supplementary material for: Analysing the mutational status of adenomatous polyposis coli (APC) gene in breast cancer
Source: Cancer Cell Int. 2016 Mar 28;16:23. doi: 10.1186/s12935-016-0297-2 (PMC4810512; doi:10.1186/s12935-016-0297-2)
Supplement: Supplementary file 1 — 10.1186/s12935-016-0297-2 HRM assays for APC exons 1, 2, 3 and 5-15. [file 12935_2016_297_MOESM1_ESM.doc]

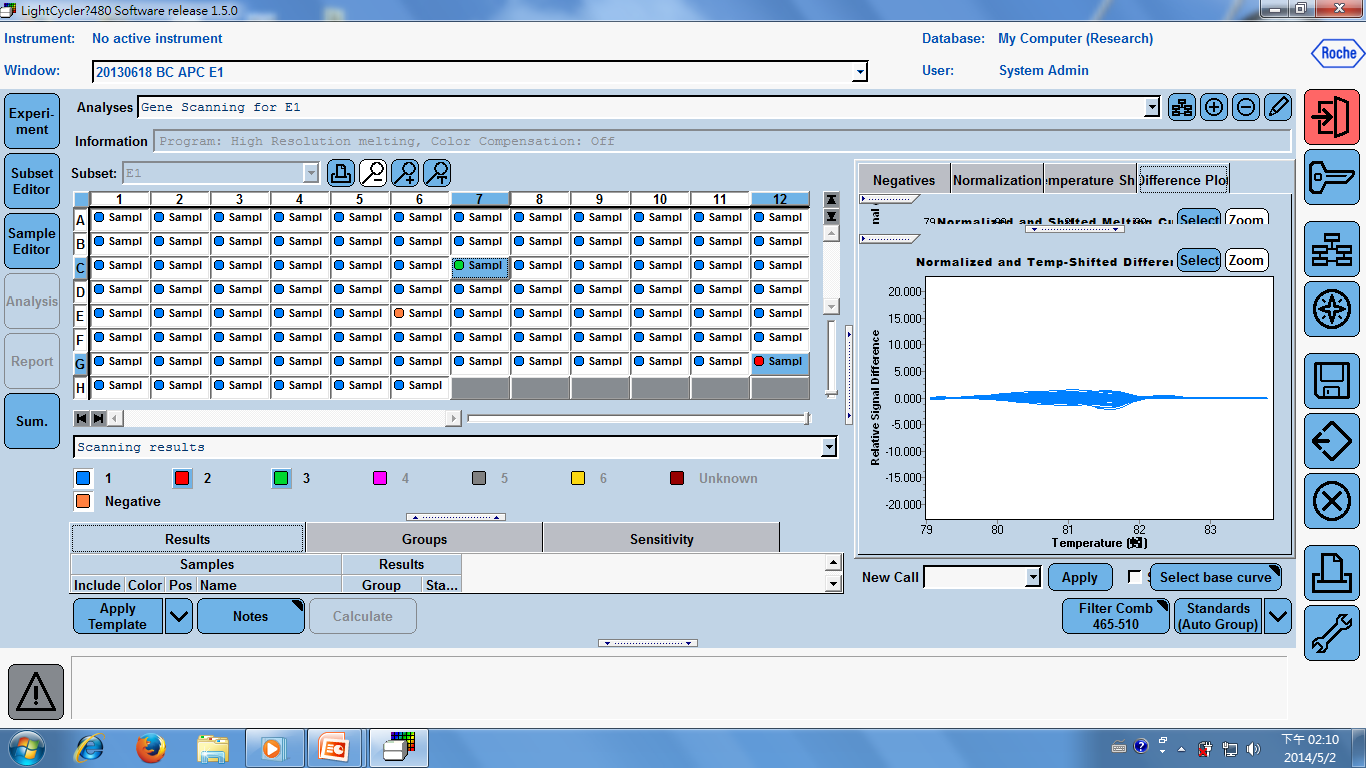


codons 1 to 45

**Figure S1:** Normalized and temperature-shifted difference plot of the HRM analysis for detecting exon 1 mutations of the *APC* gene from breast cancer patients.


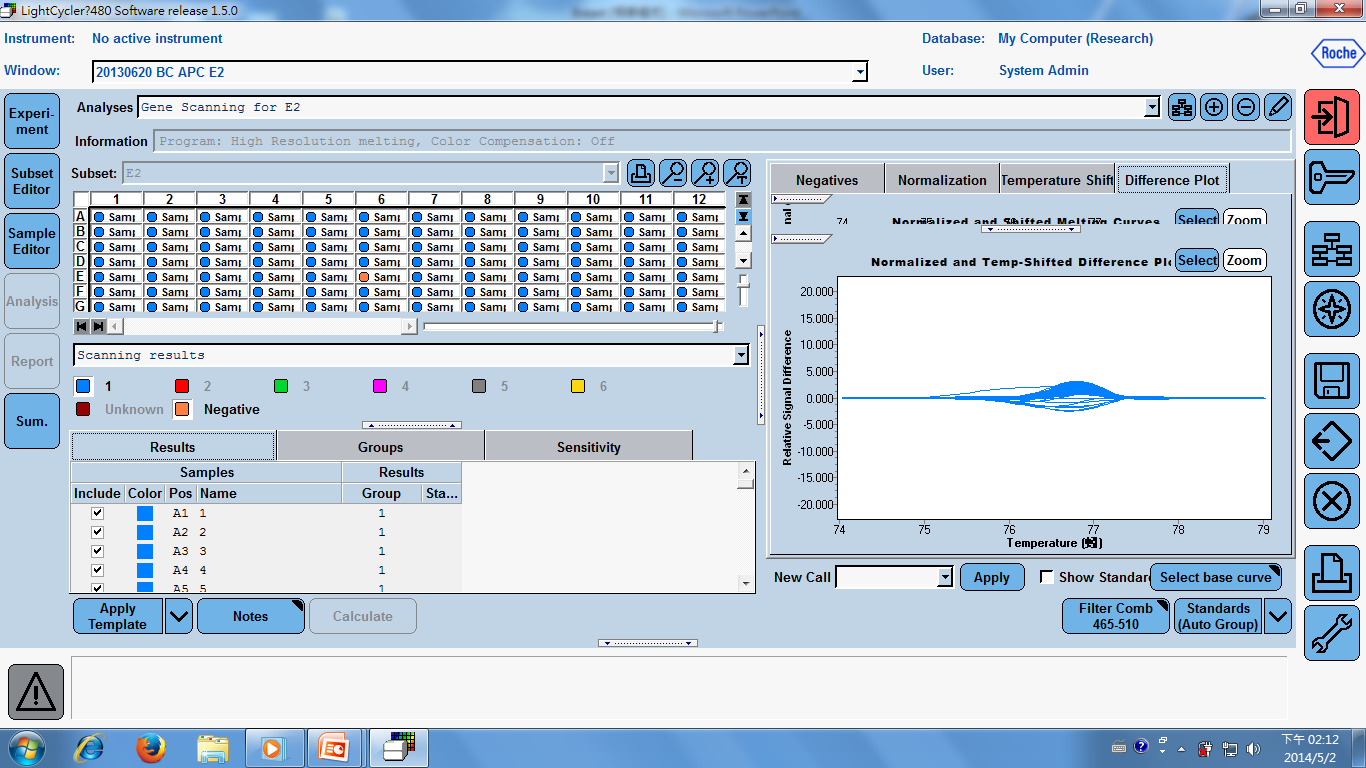


codons 46 to 74

**Figure S2:** Normalized and temperature-shifted difference plot of the HRM analysis for detecting exon 2 mutations of the *APC* gene from breast cancer patients.


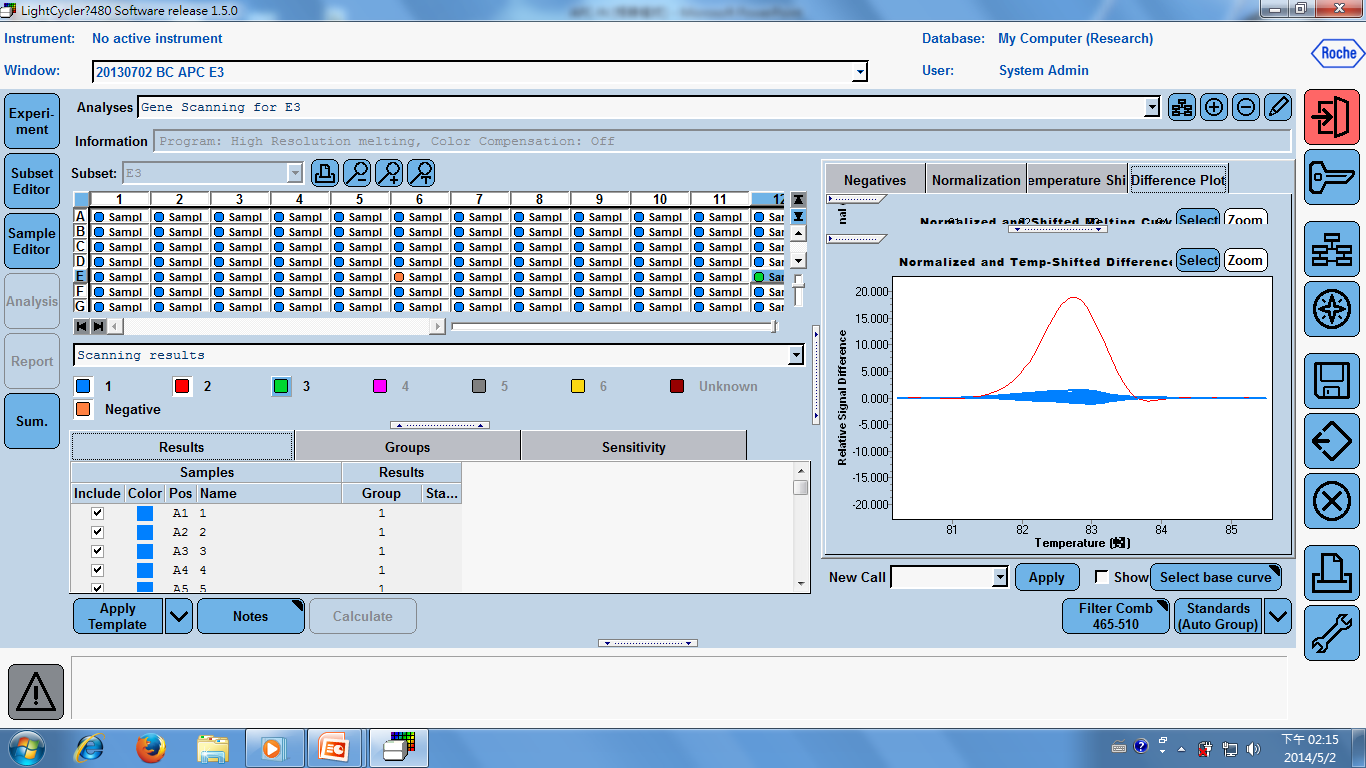


codons 75 to 141

PC

**Figure S3:** Normalized and temperature-shifted difference plot of the HRM analysis for detecting exon 3 mutations of the *APC* gene from breast cancer patients. PC: Positive Control


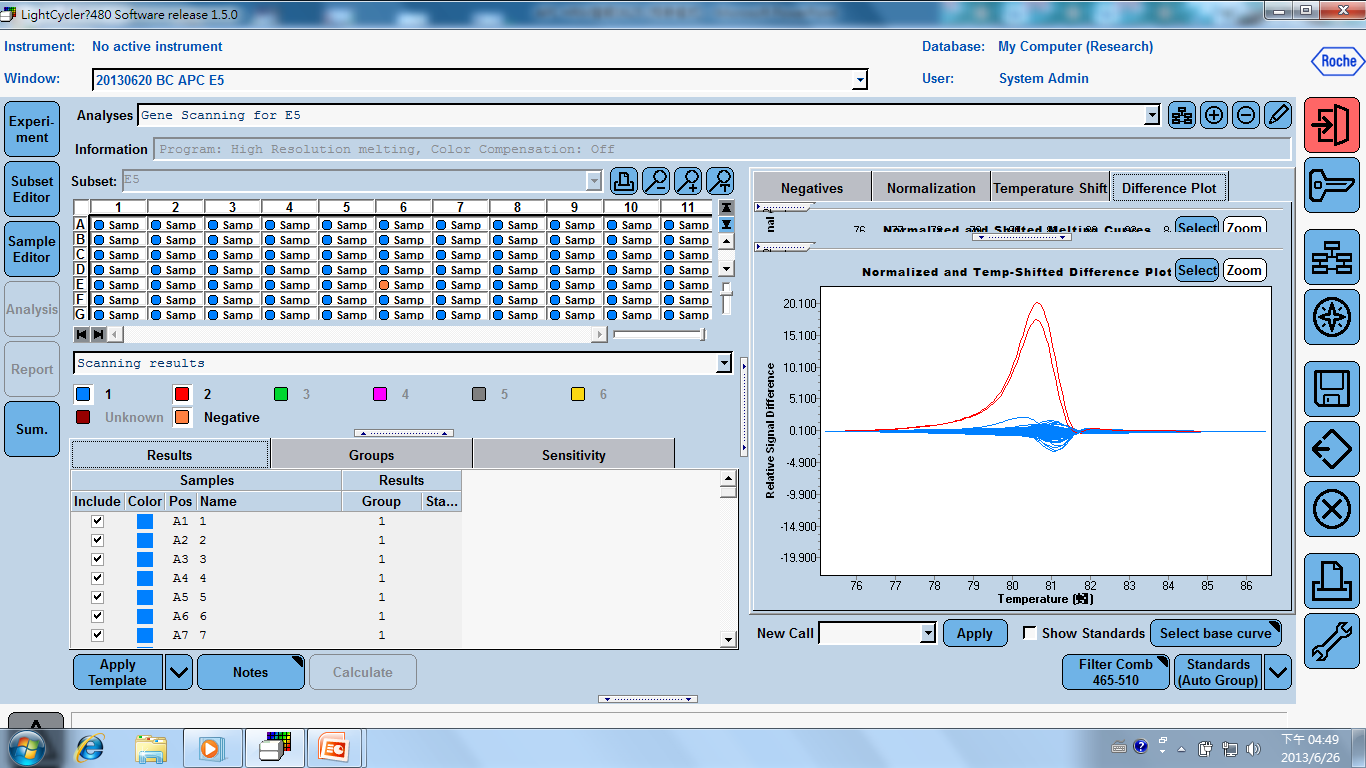


c.573T>C

PC

Wild-type

codons 178 to 215

**Figure S4:** Normalized and temperature-shifted difference plot of the HRM analysis for detecting exon 5 mutations of the *APC* gene from breast cancer patients. PC: Positive Control


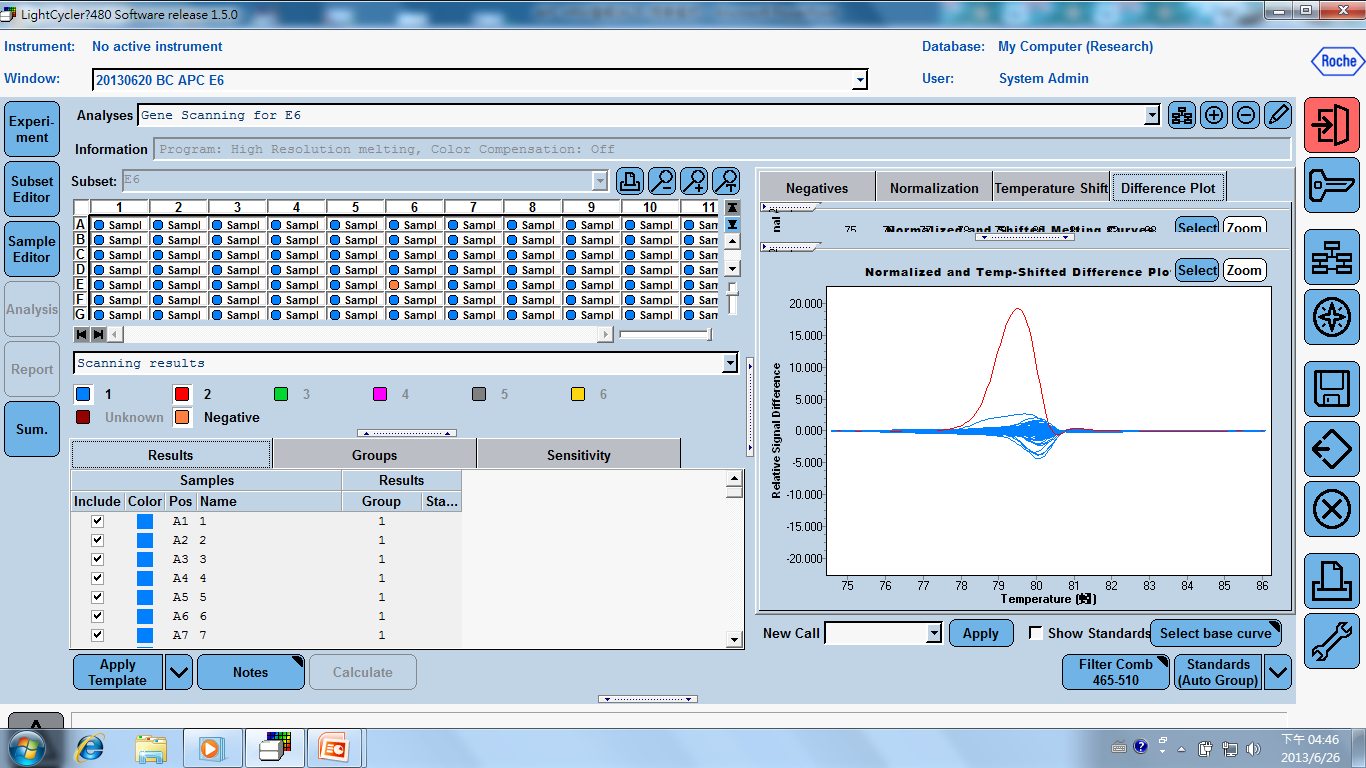


PC

codons 216 to 243

**Figure S5:** Normalized and temperature-shifted difference plot of the HRM analysis for detecting exon 6 mutations of the *APC* gene from breast cancer patients. PC: Positive Control


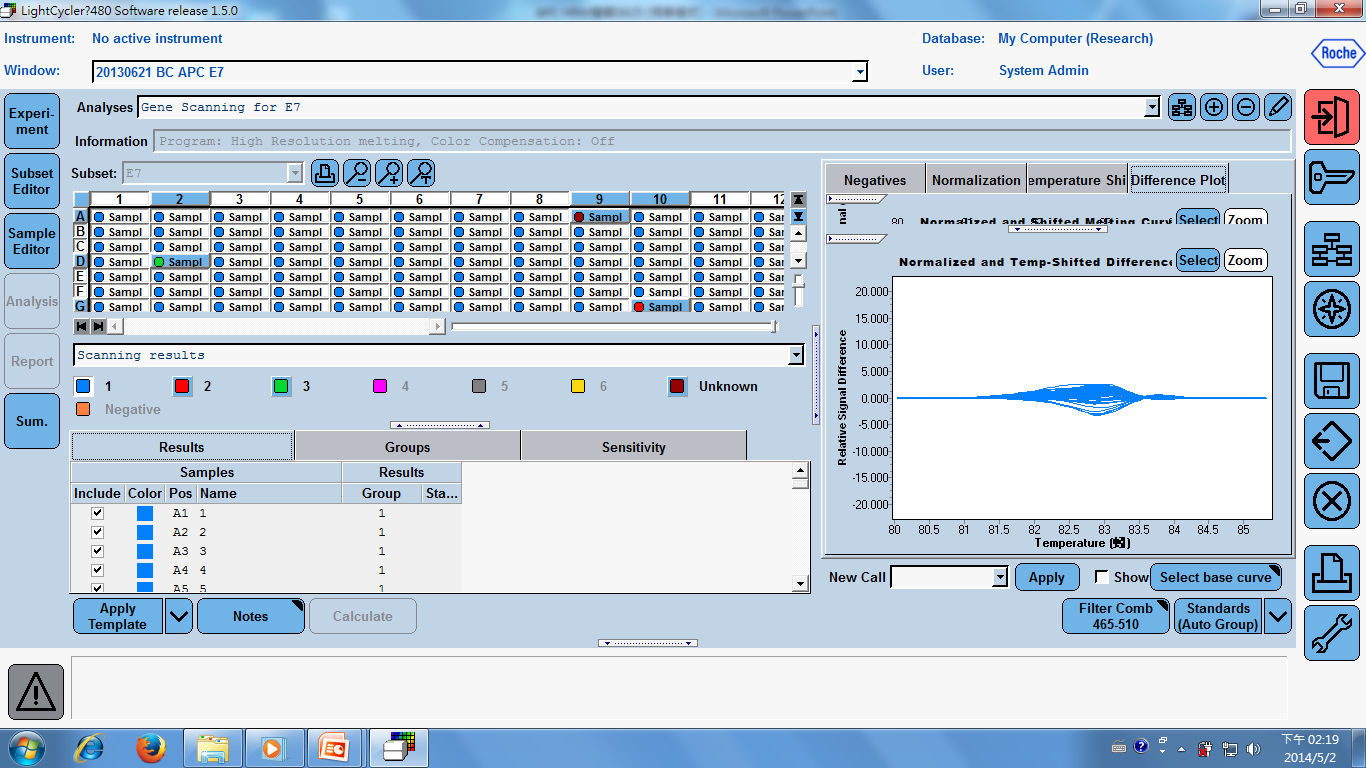


codons 244 to 278

**Figure S6:** Normalized and temperature-shifted difference plot of the HRM analysis for detecting exon 7 mutations of the *APC* gene from breast cancer patients.


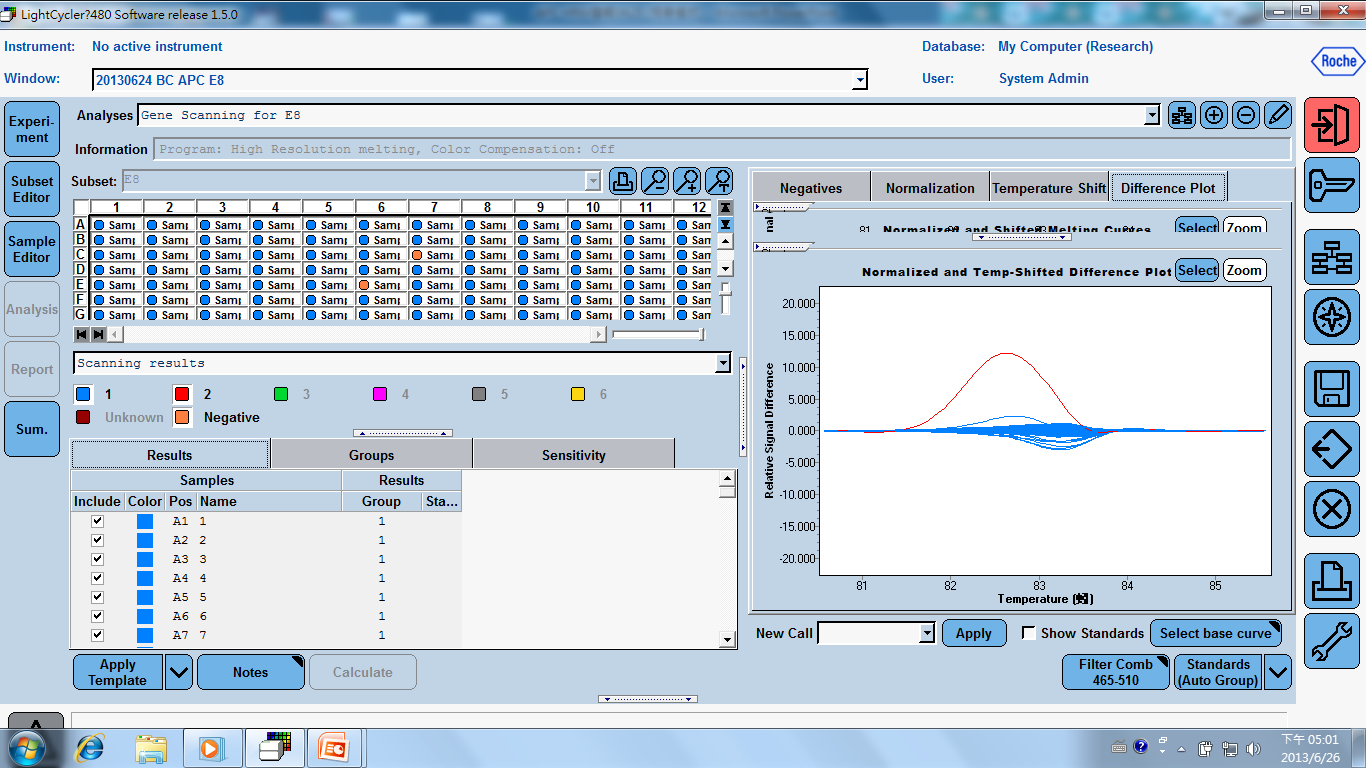


codons 279 to 311

PC

**Figure S7:** Normalized and temperature-shifted difference plot of the HRM analysis for detecting exon 8 mutations of the *APC* gene from breast cancer patients. PC: Positive Control


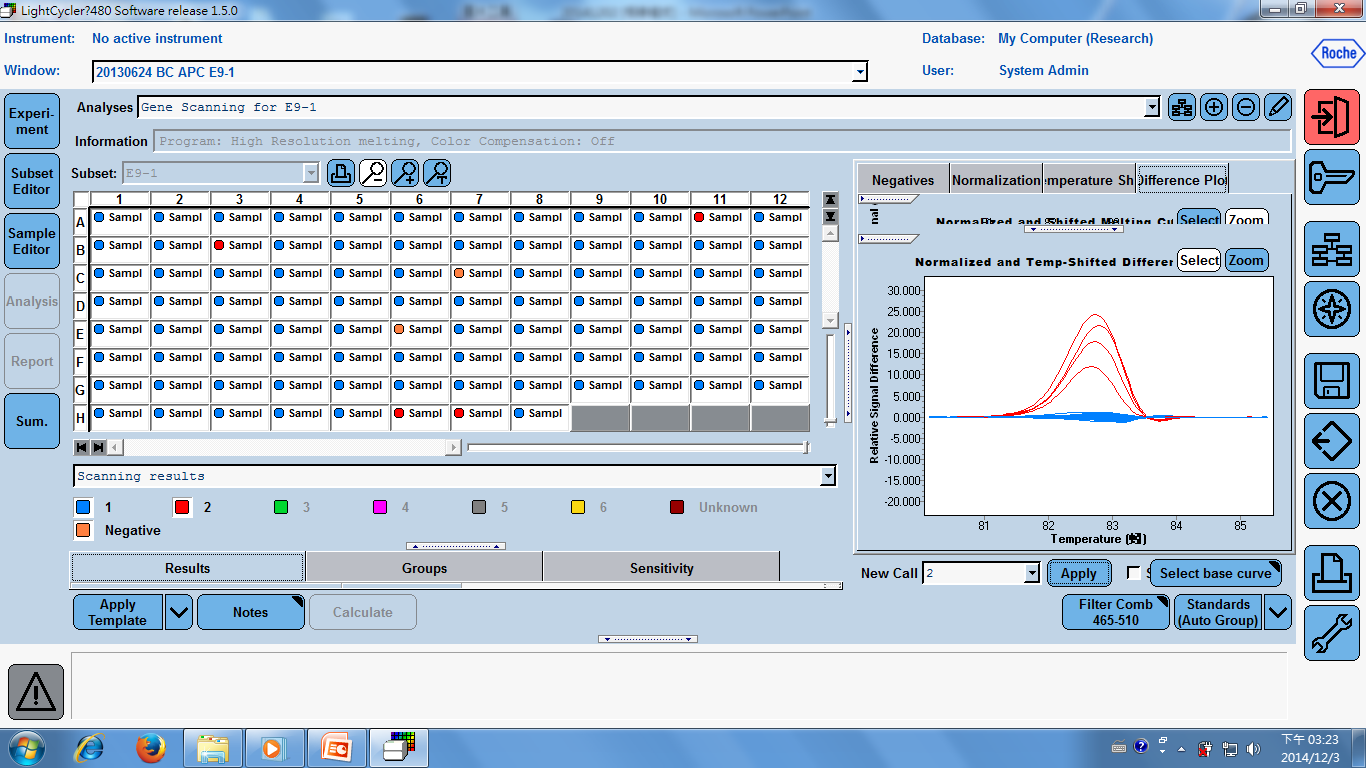


codons 351 to 438

PC

PC

codons 312 to 371

c.1005A>G


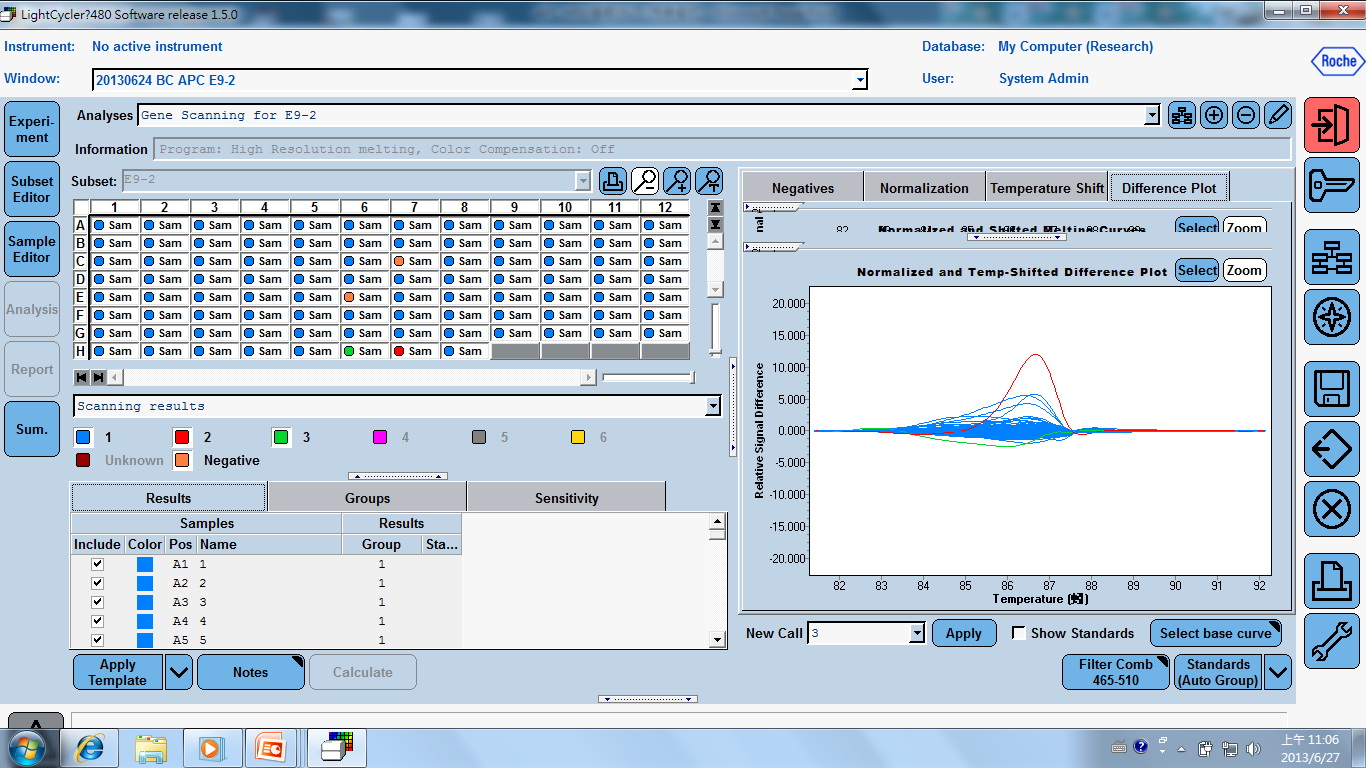


PC

**Figure S8:** Normalized and temperature-shifted difference plot of the HRM analysis for detecting exon 9 mutations of the *APC* gene from breast cancer patients. PC: Positive Control


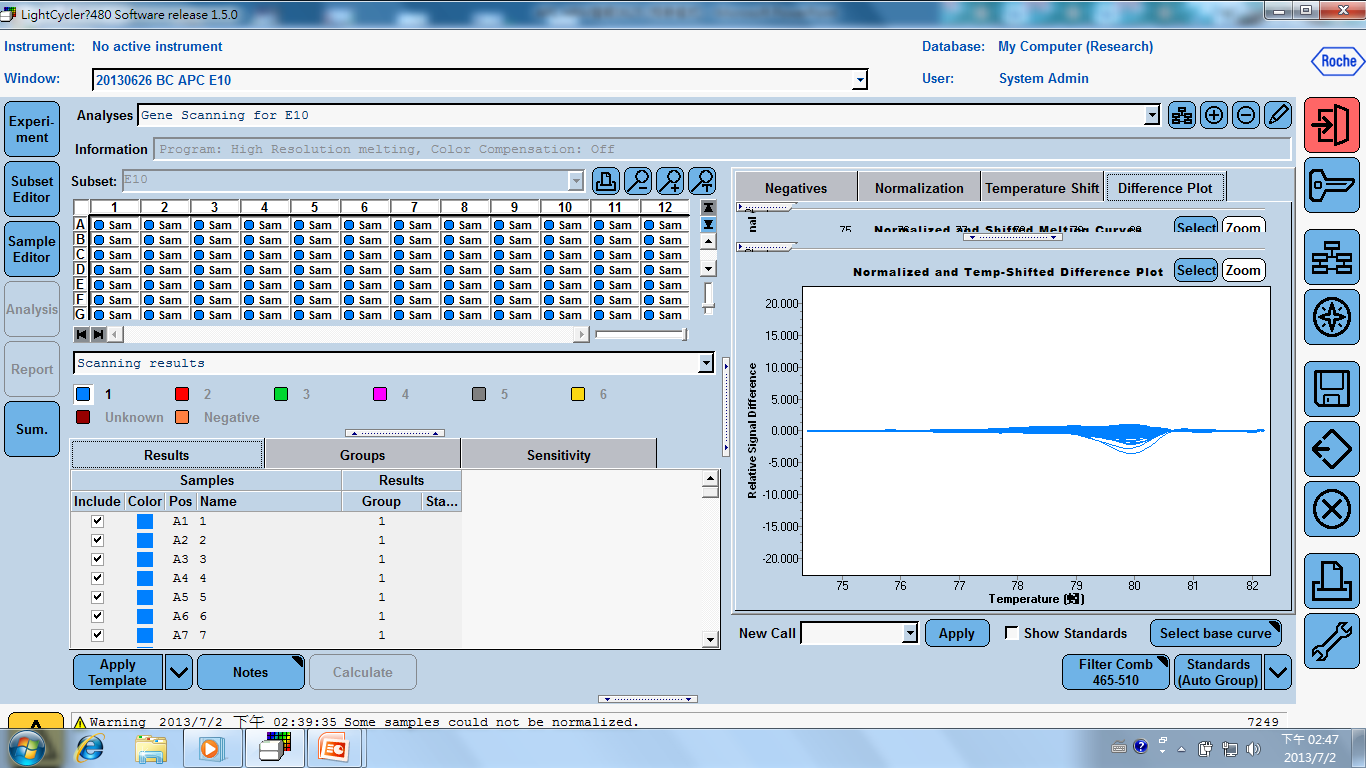


codons 439 to 470

**Figure S9:** Normalized and temperature-shifted difference plot of the HRM analysis for detecting exon 10 mutations of the *APC* gene from breast cancer patients.


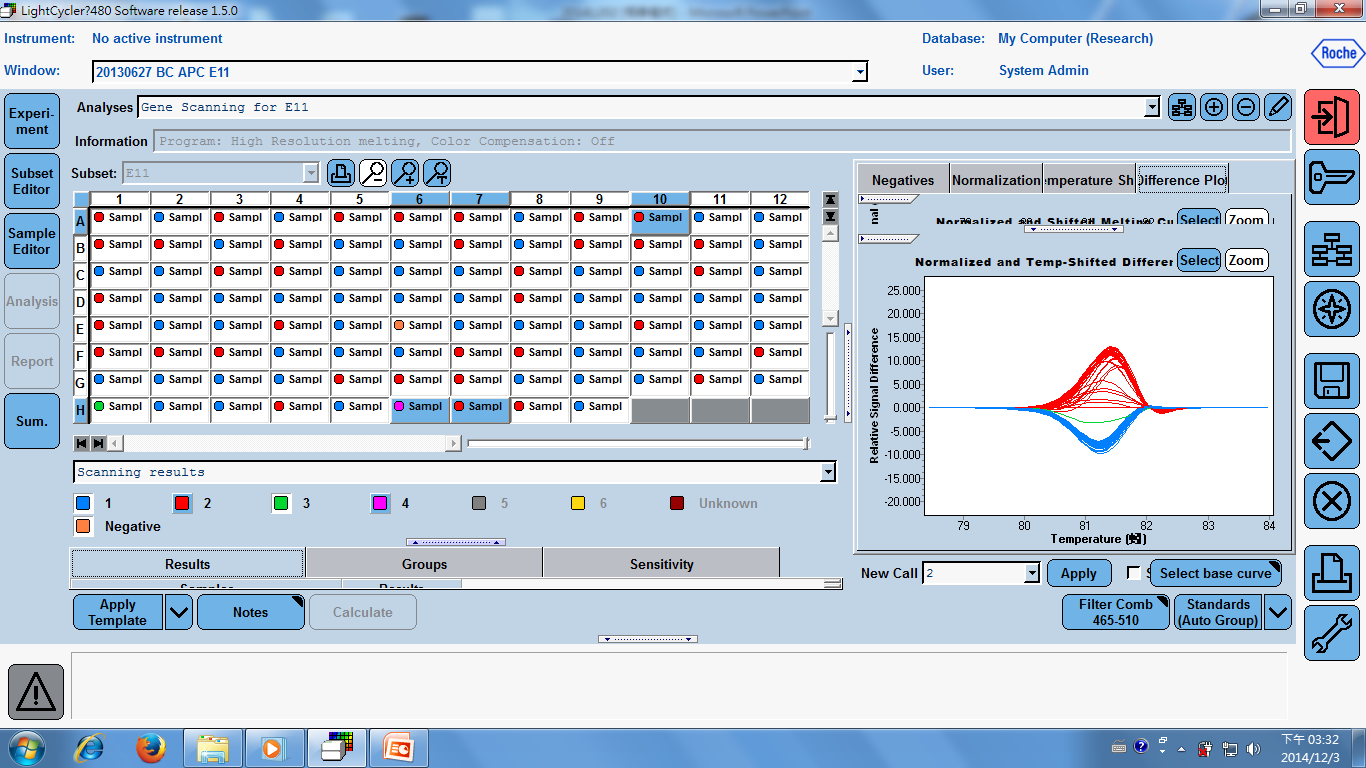


1458 (T/T or C/C)

1488 (A/A)

1458 (C/C)

1488 (A/T)

1458 (T/C)

1488 (A/A)

codons 471 to 516

codons 471 to 516


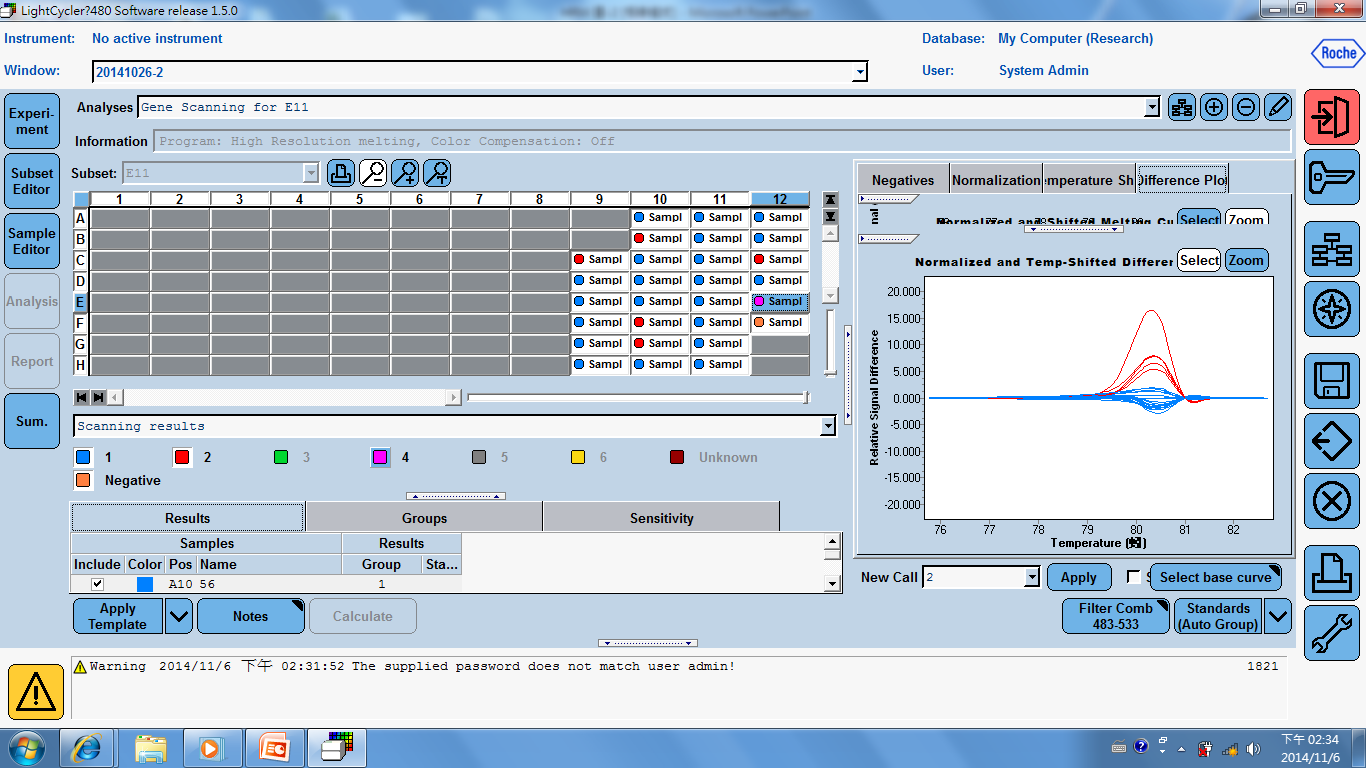


T/T

C/C

**Figure S10:** HRM assays for *APC* exon 11. (Top) Difference plot showed two different melting profiles, homozygous samples (T/T or C/C) were blue, heterozygotes (T/C) in other colors. (Bottom) Homozygous samples were discriminated by generating heterozygotes with 1:1 mixture.


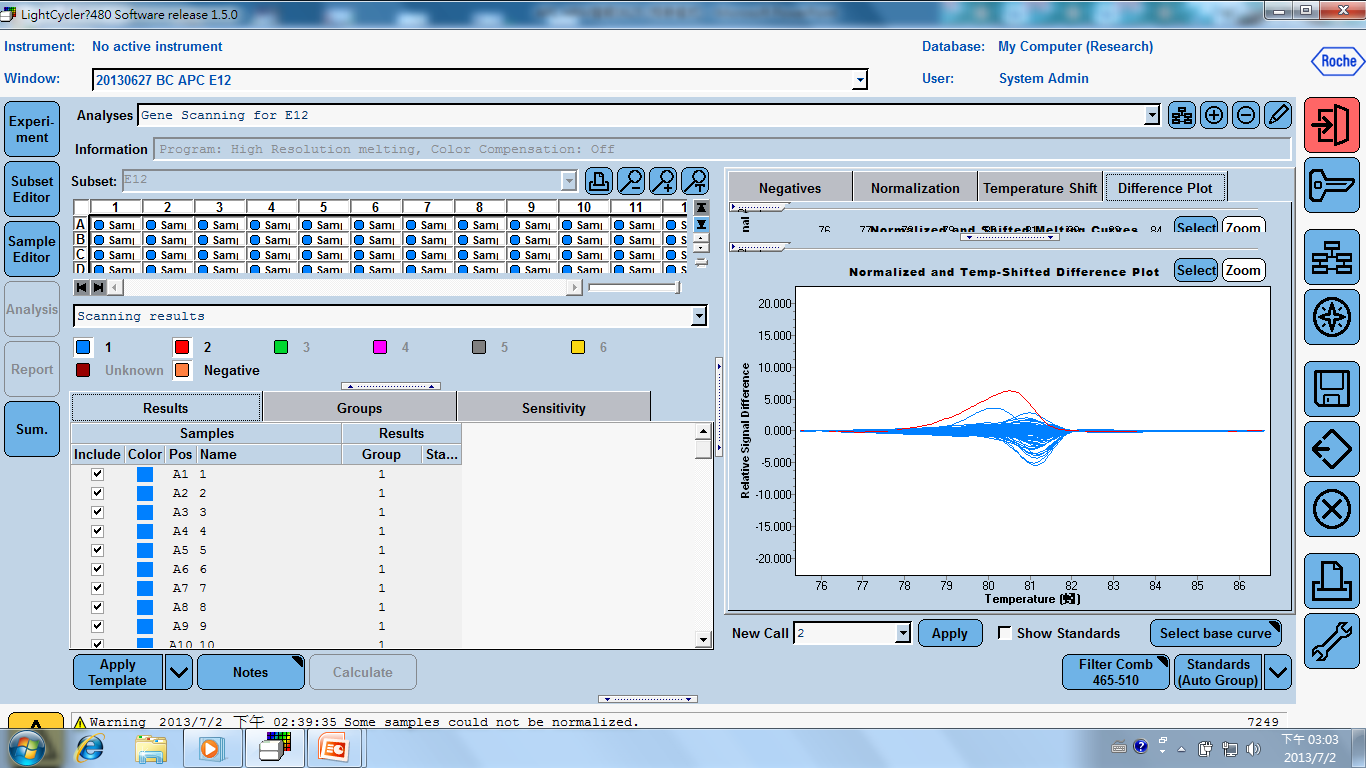


codons 517 to 542

PC

**Figure S11:** Normalized and temperature-shifted difference plot of the HRM analysis for detecting exon 12 mutations of the *APC* gene from breast cancer patients. PC: Positive Control


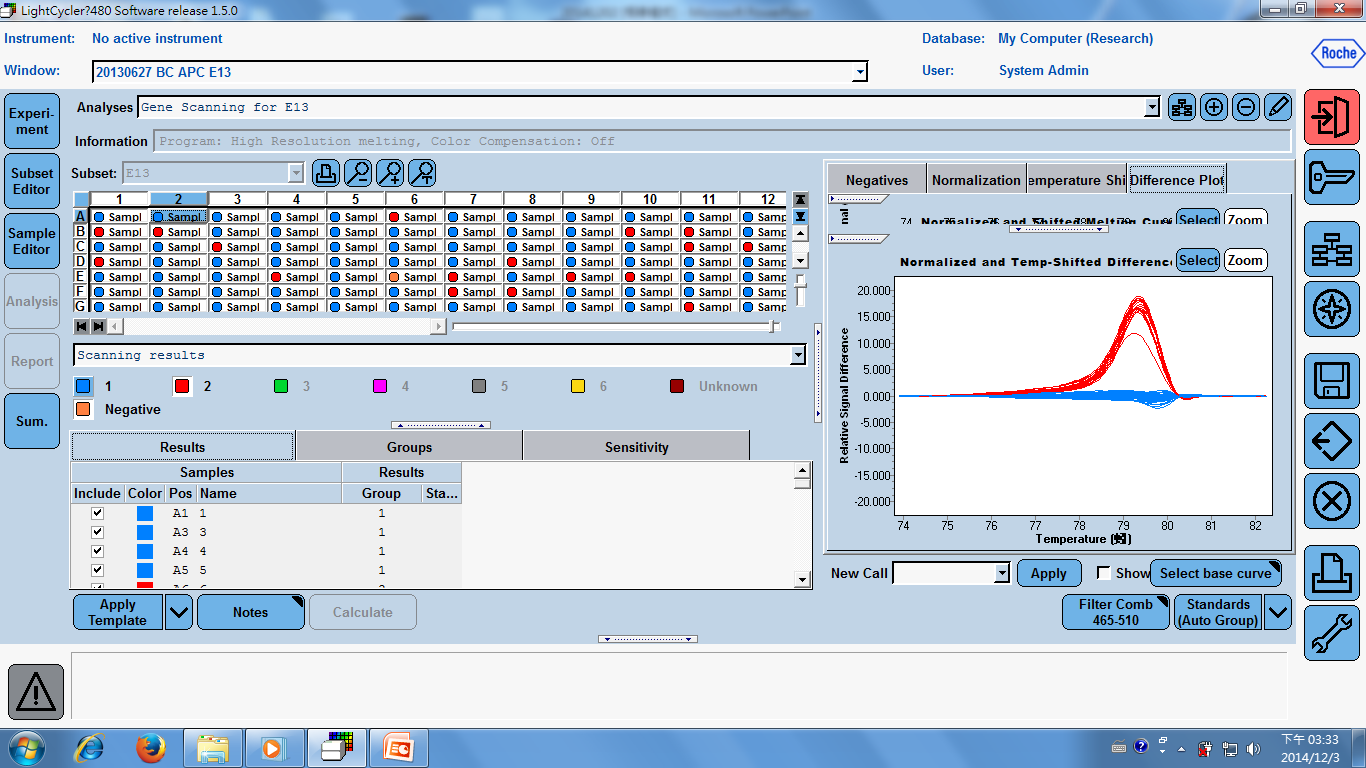


G/G or A/A

codons 543 to 569


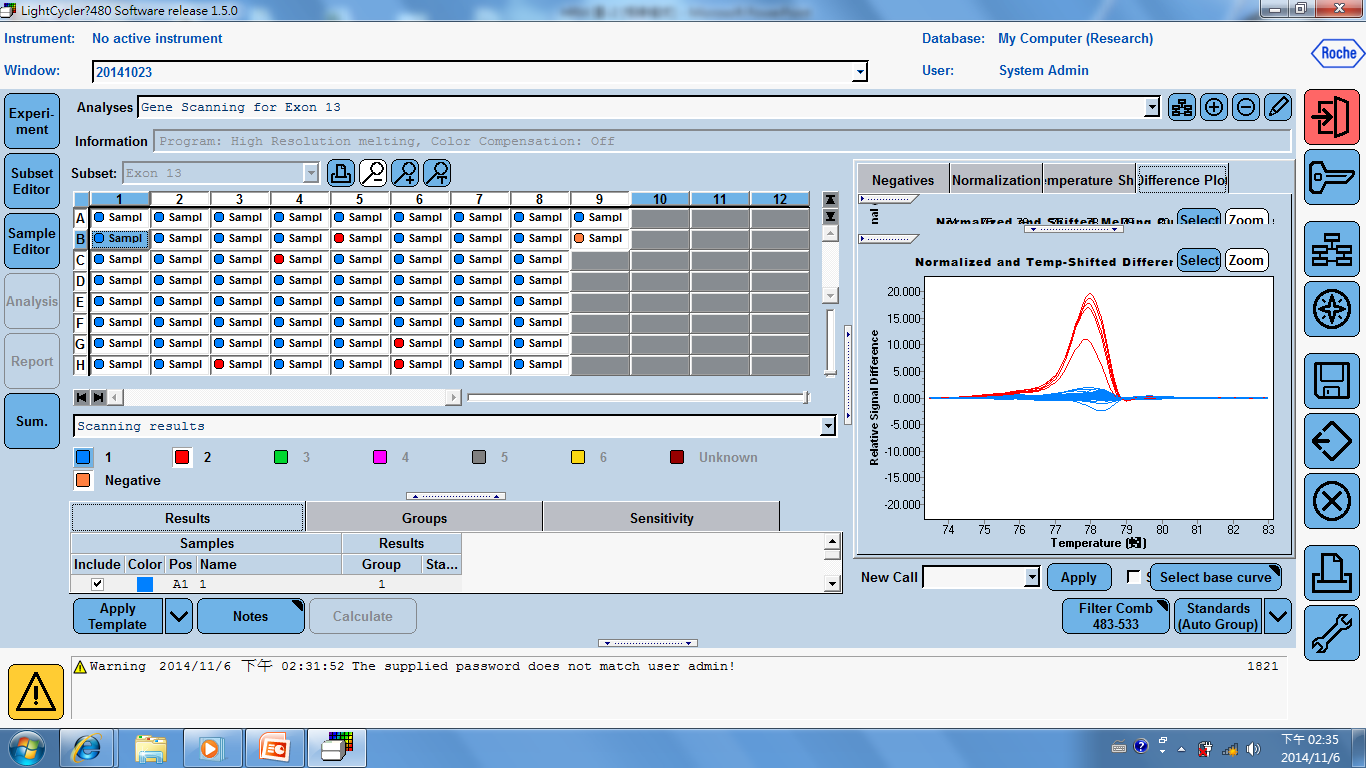


A/A

codons 543 to 569

G/G

**Figure S12:** HRM assays for *APC* exon 13. (Top) Difference plot showed two different melting profiles, homozygous samples (G/G or A/A) were blue, heterozygotes (G/A) in other colors. (Bottom) Homozygous samples were discriminated by generating heterozygotes with 1:1 mixture.


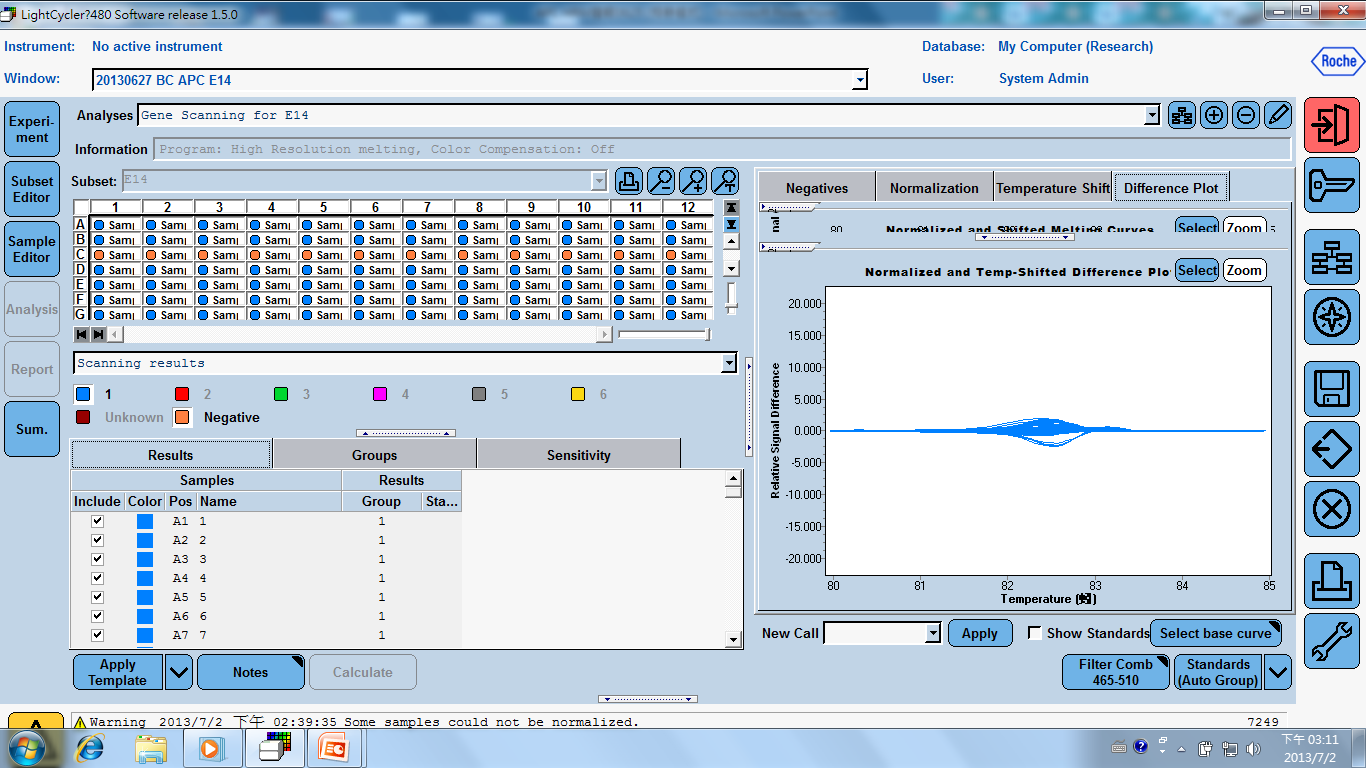


codons 570 to 641

**Figure S13:** Normalized and temperature-shifted difference plot of the HRM analysis for detecting exon 14 mutations of the *APC* gene from breast cancer patients.


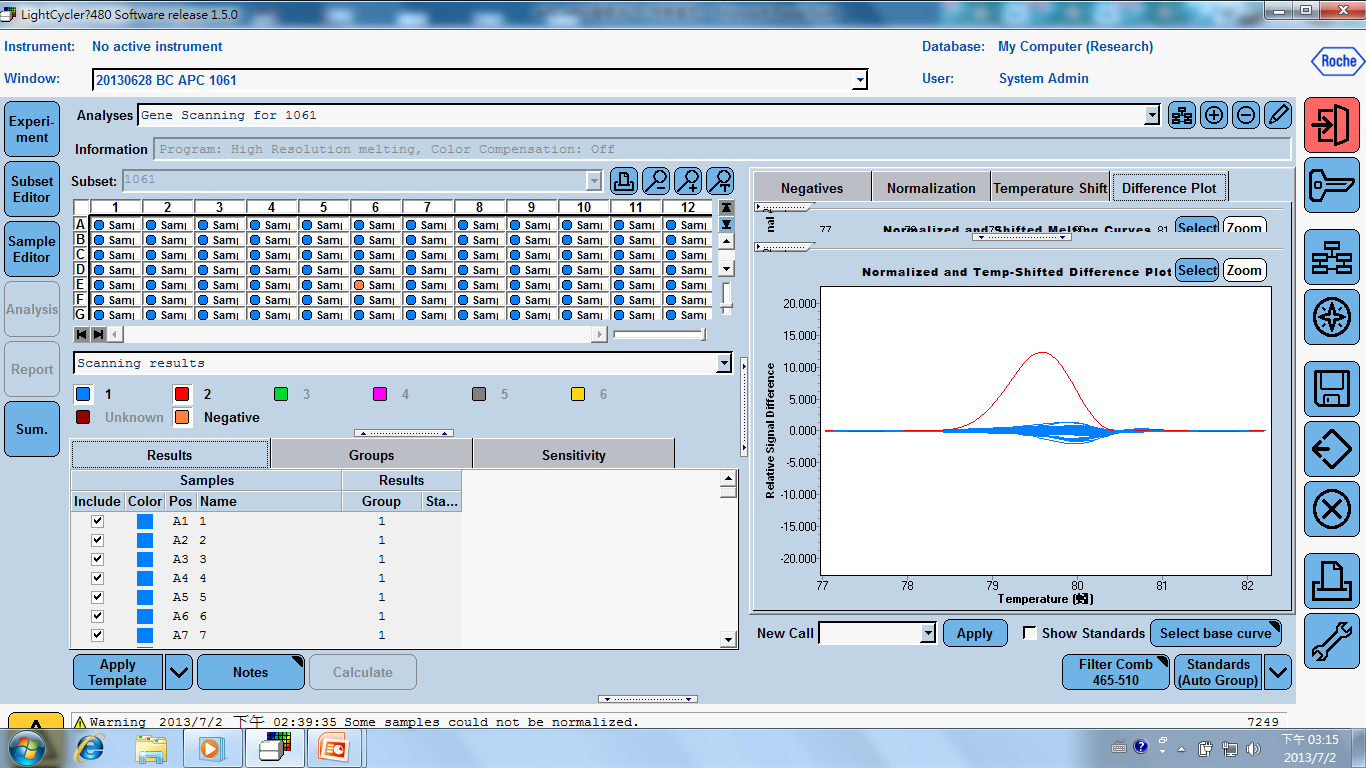


codons 1034 to 1086

PC


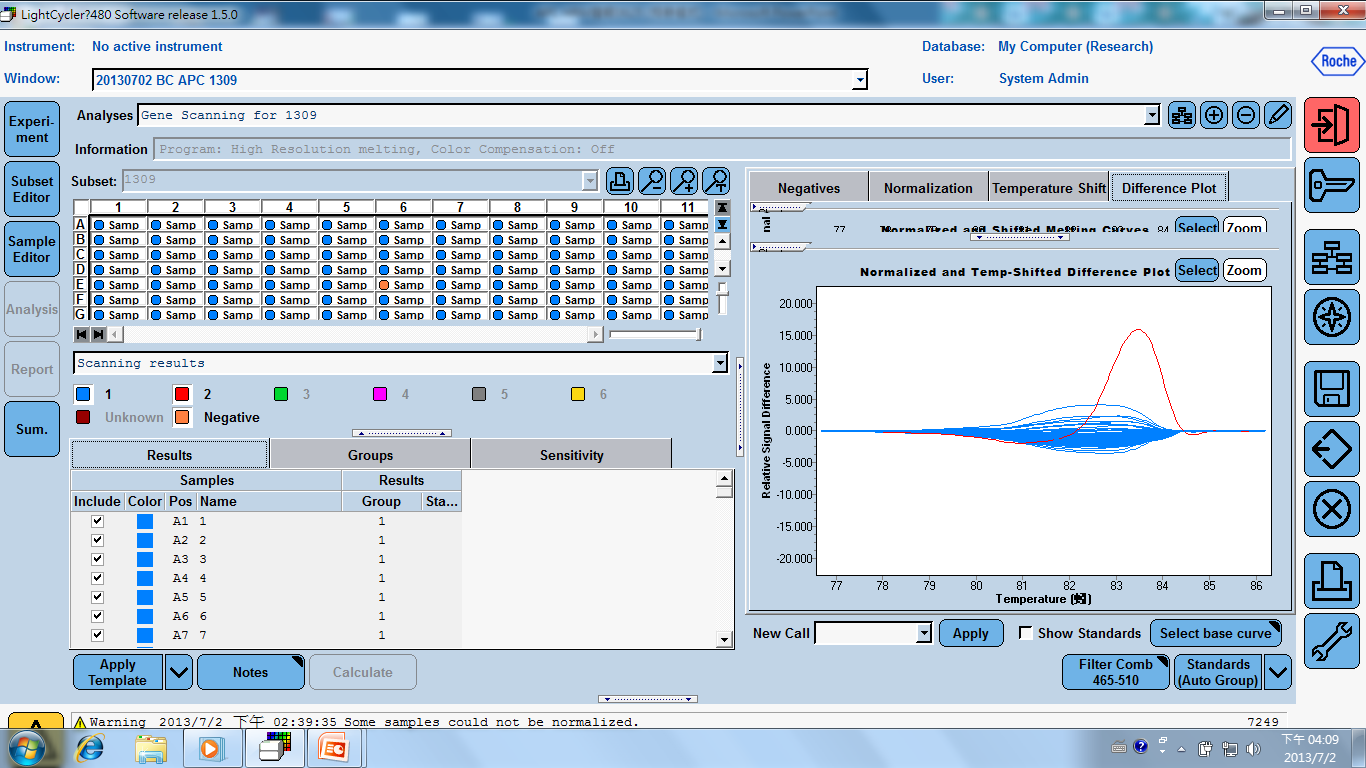


codons 1281 to 1352

PC

codons 1352 to 1441


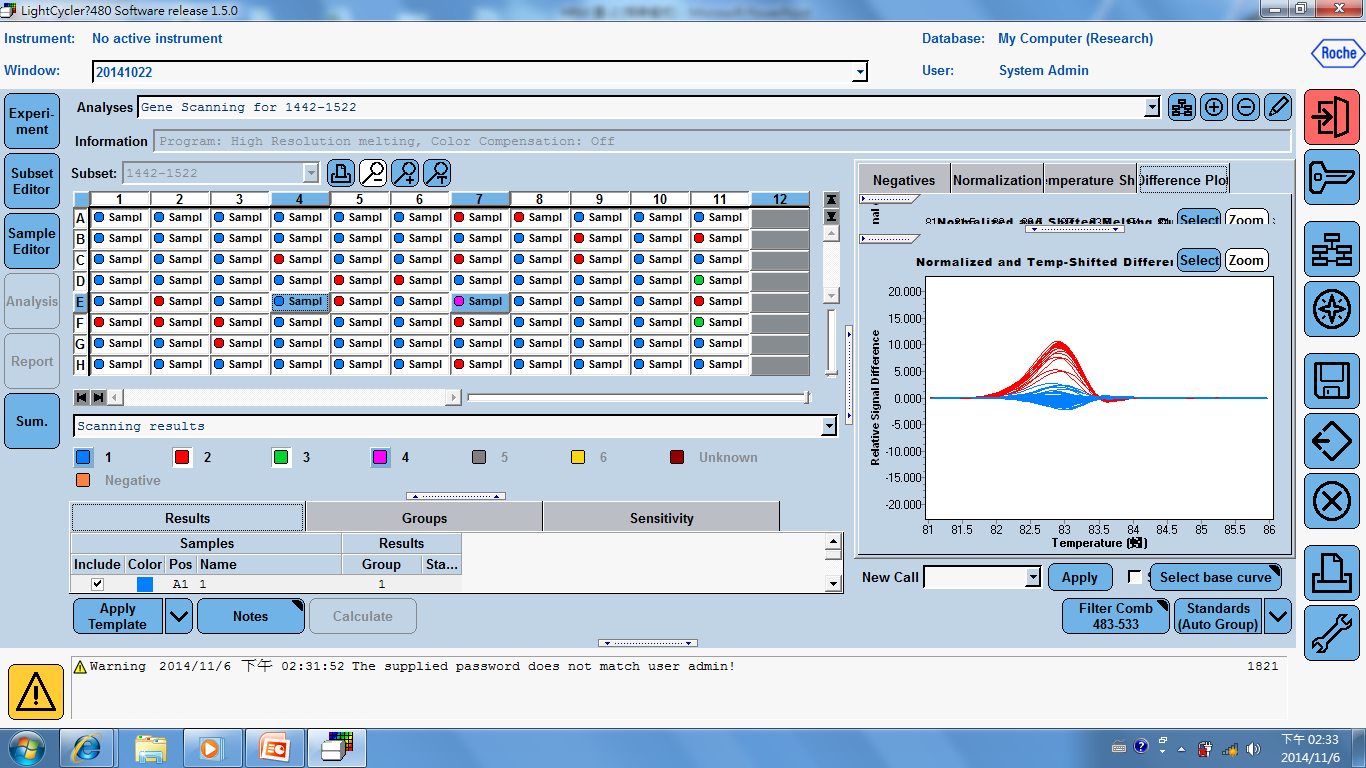


G/A

codons 1442 to 1522

codons 1442 to 1522

G/G or A/A


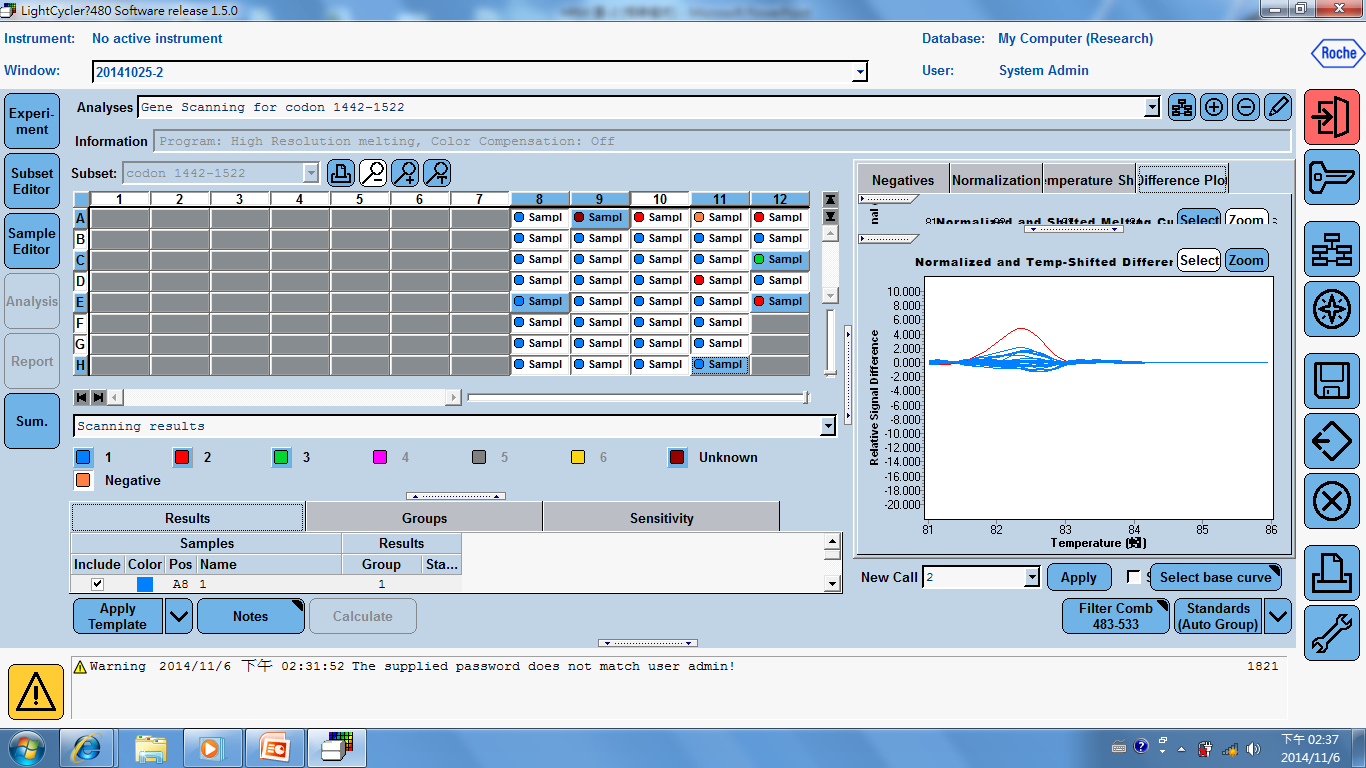


G/G

A/A


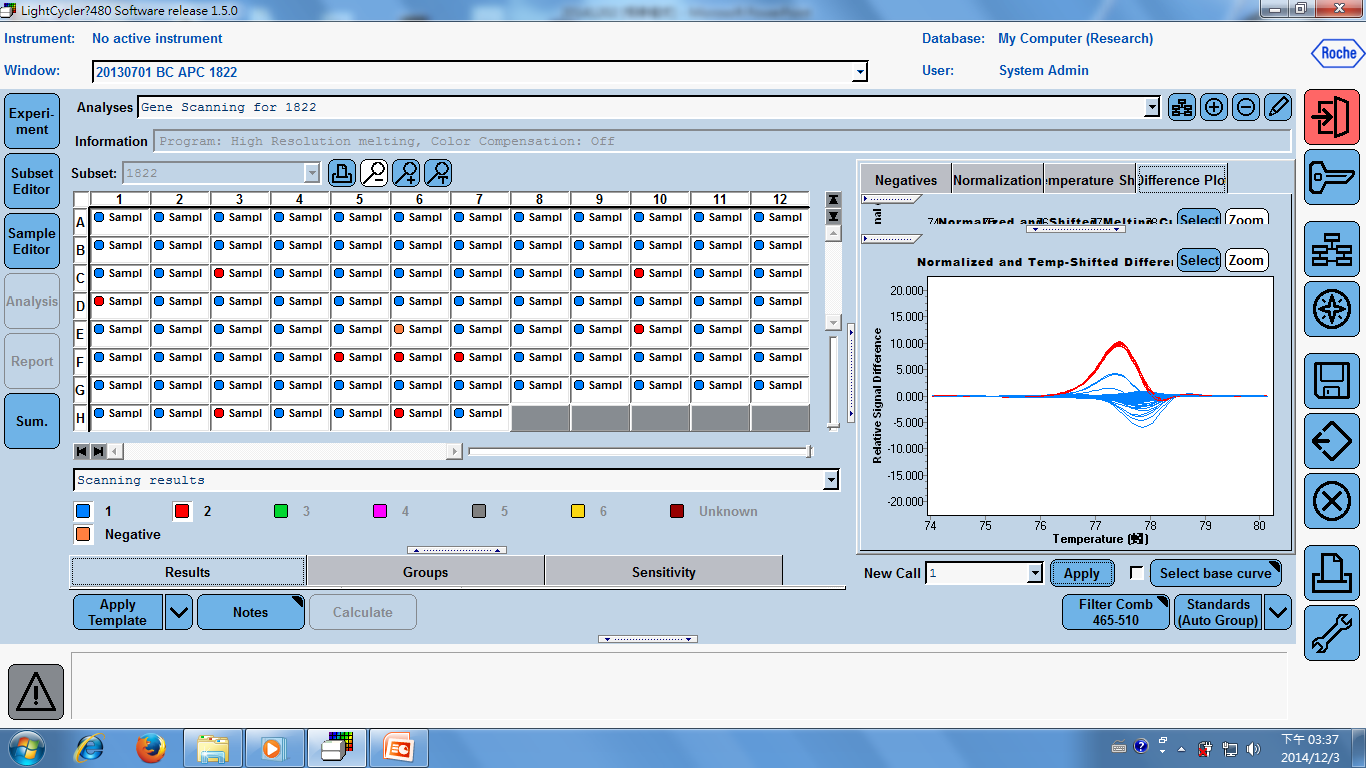


T/A

T/T or A/A

codons 1802 to 1846


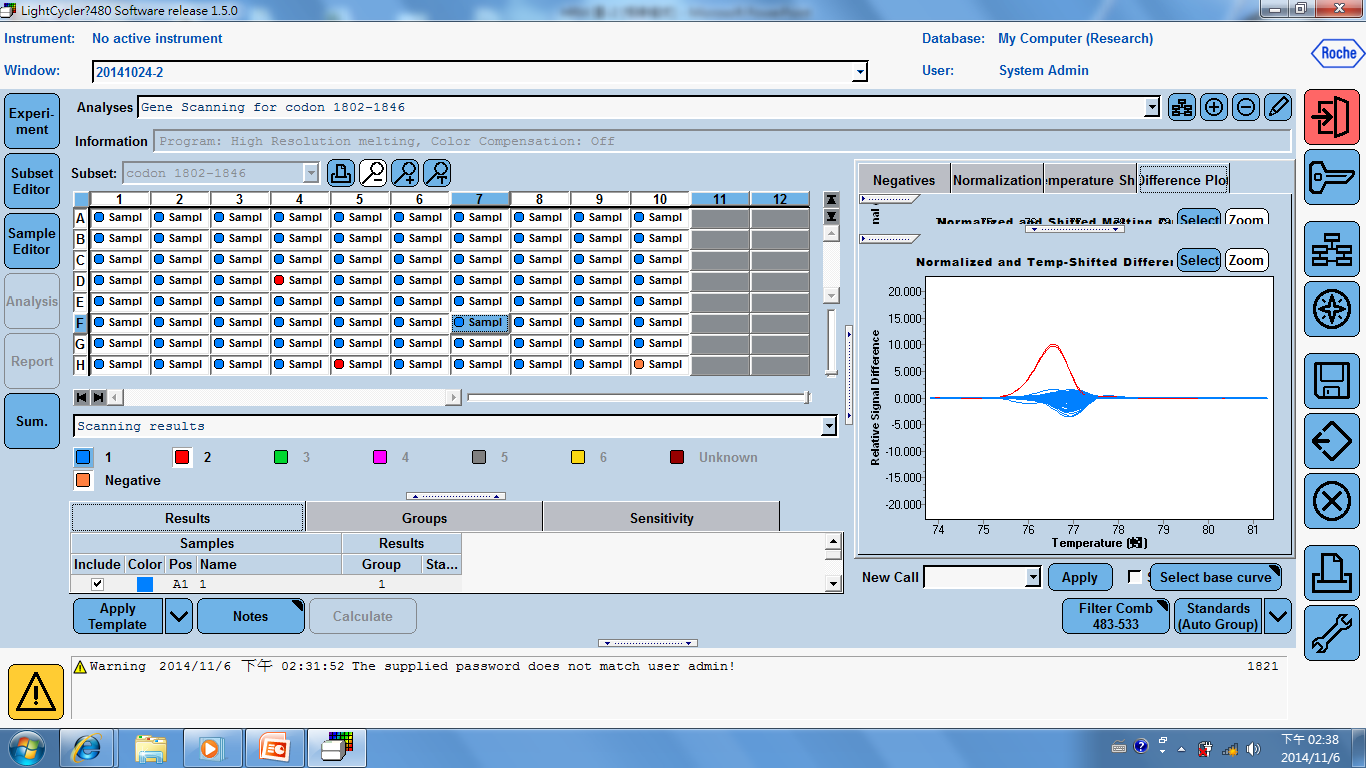


T/T

A/A

codons 1802 to 1846

**Figure S14:** Normalized and temperature-shifted difference plot of the HRM analysis for detecting exon 15 mutations of the *APC* gene from breast cancer patients.

PC: Positive Control
